# Identification of TMEM71 as a hub NLRP3-related gene suppressing malignant behavior in nasopharyngeal carcinoma via the NLRP3/Caspase-1/GSDMD signaling pathway

**DOI:** 10.1016/j.bjorl.2025.101566

**Published:** 2025-02-13

**Authors:** Dan Liu, Yuanzhou Liu, Ruixiang Cen

**Affiliations:** Huangshi Central Hospital (Affiliated Hospital of Hubei Polytechnic University), Department of Otolaryngology, Huangshi, China

**Keywords:** TMEM71, NLRP3, Caspase-1, GSDMD, Nasopharyngeal carcinoma

## Abstract

**Objective:**

NLRP3 plays a key role in cellular pyroptosis and tumor progression. However, research on NLRP3-Related Genes (NRGs) in Nasopharyngeal Carcinoma (NPC) is limited.

**Methods:**

We analyzed the GSE53819 dataset to identify genes positively correlated with NLRP3 mRNA and downregulated in NPC tumors, termed NRGs. Gene Ontology (GO) and Kyoto Encyclopedia of Genes and Genomes (KEGG) analyses were used to characterize their biological functions. Validation was performed using the GSE64634 and GSE102349 datasets. The GSE102349 dataset was used to evaluate the impact of NRGs on the Progression-Free Survival (PFS) and their association with immune cell infiltration. A cohort of 421 NPC patients from a local hospital underwent multivariate Cox regression to assess the prognostic significance of hub NRGs. Cellular experiments further investigated the role of hub NRGs in NPC.

**Results:**

In the GSE53819 dataset, 26 NRGs were identified, correlated with NLRP3 expression, and downregulated in tumor tissues. GO and KEGG analyses linked these 26 NRGs to the inflammasome complex. TMEM71, identified in the GSE64634 and GSE102349 datasets, was downregulated in tumor tissues and positively correlated with NLRP3 expression. It was the only NRG with prognostic value, with higher expression correlating with improved PFS. Immune cell infiltration analysis showed significant differences between high and low TMEM71 expression groups (e.g., naïve B cells). Local analysis confirmed that positive TMEM71 expression in tumor serves as an independent prognostic marker for NPC (HR = 0.53, 95% CI 0.366‒0.780). in vitro, TMEM71 activation of the NLRP3/caspase-1/GSDMD pathway suppressed malignant behaviors in NPC cell.

**Conclusion:**

TMEM71 may serve as a prognostic biomarker for NPC and influence immune cell infiltration. Its overexpression could exert anticancer effects via the NLRP3/caspase-1/GSDMD pathway, highlight its potential as a therapeutic target in NPC.

## Highlights

• Identified 26 key NRGs in NPC tumors.
• TMEM71 linked to immune cell infiltration in NPC.
• TMEM71 as a prognostic factor in NPC.
• TMEM71 inhibits NPC malignant behavior via NLRP3/caspase-1/GSDMD pathway.

## Introduction

Nasopharyngeal Carcinoma (NPC) is a malignant of the head and neck, strongly linked to the Epstein-Barr Virus (EBV), and predominantly affects populations in East and Southeast Asia. In 2018, these regions accounted for 70% of the global 129,000 NPC cases.^1^ Despite treatment advances, approximately 30% of NPC patients have a median survival of 10–36 months, with the five-year survival rate for stage IV patients remaining below 40%.^2^, ^3^, ^4^ Additionally, 73.1% of cases are diagnosed at advanced stages due to vague early symptoms, highlighting the need for improved diagnostic and therapeutic strategies.^5^

Pyroptosis, a form of programmed cell death characterized by the activation of inflammatory responses, has garnered significant attention in cancer research for its critical role in modulating tumor progression.^6^, ^7^ This process is triggered by NLRP3 inflammasome activation, leading to Caspase-1 cleavage and the activation of GSDMD. As a key factor in pyroptosis, the role of NLRP3 in cancer has been widely investigated.^8^ In NPC, previous studies have explored the potential involvement of the NLRP3 inflammasome.^9^, ^10^ Our prior work also identified the NLRP3/caspase-1/GSDMD pathway as a key biomarker for predicting NPC recurrence and metastasis.^11^ However, the regulatory mechanisms of NLRP3 in NPC, especially its upstream activators and downstream effects, remain largely unclear.

TMEM71, a transmembrane protein, has been identified as a potential biomarker and therapeutic target in central nervous system metastasis in acute lymphoblastic leukemia.^12^ Although no studies have directly linked TMEM71 to NLRP3, other members of the TMEM family, have been shown to regulate pyroptosis by modulating intracellular calcium levels, promoting inflammasome formation, and activating caspase-1.^13^, ^14^ TMEM71 may participate in the activation of the inflammasome through similar mechanisms, thus playing a role in NPC progression. Therefore, investigating the interaction between TMEM71and NLRP3 may reveal its specific regulatory role in NPC progression and open up new therapeutic avenues.

In this study, we employed bioinformatics to identify TMEM71 as a key gene associated with the pyroptosis regulator NLRP3, clarifying its expression patterns, relationships with immune cell infiltration, and its potential diagnostic and prognostic value in NPC. Cellular experiments further elucidated the role of TMEM71, particularly within the NLRP3/Caspase-1/GSDMD pathway in NPC. Our findings provide insights for early diagnosis, personalized treatment, and the development of novel molecular targets for NPC.

## Methods

### Data acquisition and processing

The transcriptome data of NPC were obtained from the Gene Expression Omnibus (GEO) database, including GSE64634, GSE53819, and GSE102349 datasets. Progression-Free Survival (PFS) follow-up data were specifically available in the GSE102349 dataset. Additionally, a retrospective analysis was performed on 421 NPC patients from Huangshi Central Hospital between December 2014 and January 2020, with follow-up until March 2023. Eligible patients were aged ≥18 with pathologically confirmed primary tumors. Exclusion criteria included distant metastasis, severe chronic diseases, refusal of treatment, or incomplete data. All patients were treatment-naive before biopsy, with a minimum follow-up of three years. The relationship between NPC patients and survival was analyzed using the ‘survminer’ (v0.4.9) and ‘survival’ (v3.5-5) packages. The study was approved by the ethics committee of Huangshi Central Hospital (Approval No. 2024-27). and exempt from informed consent due to the absence of private patient information or commercial interests.

### Acquisition of NLRP3-related genes (NRGs)

In the GSE53819 dataset, transcriptomic data from 18 tumor samples were analyzed. Genes positively correlated with NLRP3 expression (correlation coefficient >0.6, *p*-value <0.001) were initially selected. Differentially expressed genes between 18 normal nasopharyngeal tissues and 18 NPC tissues were identified using the 'limma' package (v3.58.1) with thresholds of |logFC| ≥1 and adjusted *p*-value <0.05. A total of 26 NRGs were identified by intersecting genes from both the correlation and differential expression analyses using the 'venn' package (v1.12).

### Biological function analysis

Gene Ontology (GO) and Kyoto Encyclopedia of Genes and Genomes (KEGG) enrichment analyses were performed on 26 NRGs, with pathways considered significantly enriched if the Benjamini-Hochberg corrected p-value and FDR (q-value) were < 0.05. These analyses were conducted using the ‘clusterProfiler’ (v4.8.1), ‘org.Hs.eg.db’ (v3.17.1), and ‘enrichplot’ (v1.20.0) packages.

### Immune cell infiltration analysis

To evaluate immune cell infiltration in NPC, the CIBERSORT algorithm was applied to 113 NPC samples from the GSE102349 dataset. Based on the median TMEM71 expression level (1.596), the samples were divided into high (57 samples) and low (56 samples) TMEM71 expression groups, and differences in immune cell infiltration between the two groups were compared.

Molecular docking and structural predictionThe amino acid sequences of TMEM71 and NLRP3 were obtained from the UniProt database (Q6P5 × 7/Q96P20). Protein tertiary structure prediction was performed using AlphaFold 3 (https://golgi.sandbox.google.com/). Molecular docking between TMEM71 and NLRP3 was conducted using the HDOCK server (http://hdock.phys.hust.edu.cn/), and the docking results were visualized using PyMOL (v2.5.0).

## IHC

Sections (4 μm) from 421 NPC tumor paraffin-embedded samples were deparaffinized with xylene and hydrated through a graded ethanol series. Antigen retrieval was achieved by microwaving sections in sodium citrate buffer (Ph = 6.0) for IHC for 10 min each. After blocking with 5% goat serum for 30 min at room temperature, primary antibodies (TMEM71 at 1:50 dilution, Abcam, Shanghai, China) were applied and incubated overnight at 4 °C. After washing the sections three times with PBS for 5 min each, the corresponding secondary antibody (HRP-conjugated goat anti-rabbit antibody, 1:200 dilution, Abcam) was applied and incubated at room temperature for 1 h. Signal amplification was performed using an SP staining kit (Shanghai Enzyme-linked Biotechnology Co., Ltd.). Finally, sections were mounted, sealed, and staining intensity was scored from 0 (no staining) to 12 (strong positive), based on the proportion and intensity of stained cells.

### Immunofluorescence staining

Tissue sections were dewaxed and subjected to antigen retrieval according to the IHC protocol. Subsequently, sections were blocked with 5% Bovine Serum Albumin (BSA) or normal goat serum at room temperature for 1 h to prevent non-specific binding. The sections were then incubated with primary antibodies: TMEM71 (1:100, Abcam), NLRP3 (1:100, Abcam), and CK5/6 (1:100, Abcam) overnight at 4 °C. On the following day, sections were incubated with the corresponding secondary antibodies: TMEM71 secondary antibody was TRITC (1:500, Abcam), NLRP3 secondary antibody was Alexa Fluor 488 FITC (1:500, Abcam), and CK5/6 secondary antibody was Alexa Fluor 594 (1:500, Abcam) for 1 h at room temperature. TMEM71 displayed red fluorescence, NLRP3 showed green fluorescence, and CK5/6 showed pink fluorescence. DAPI (1:5000, Abcam) was used for counterstaining, incubated at room temperature for 5 min to label the nuclei. Afterward, the sections were washed with PBS three times, each for 5 min. Finally, sections were mounted with an anti-fluorescence quenching mounting medium and stored in the dark. Fluorescence images were captured using a fluorescence microscope (Olympus, FV1000, Tokyo, Japan), and at least three images were taken for each slide to document the fluorescence signals of TMEM71, NLRP3, and CK5/6, as well as their co-localization.

### Cell culture

The HNE-2 human NPC cell line, obtained from the American Type Culture Collection (ATCC), was thawed in a 37 °C water bath, transferred to a 15 mL centrifuge tube with pre-warmed medium, and centrifuged at 250×g for 5 min. The supernatant was discarded, and cells were resuspended in 1 mL of RPMI-1640 medium (Shanghai Chuanqiu Biotechnology Co., Ltd). Cells were cultured in DMEM containing 10% fetal bovine serum and 1% streptomycin-penicillin (Chuanqiu), and maintained in a constant temperature incubator with medium changes every two days until 80% confluence was achieved.

### Plasmid transfection and grouping

Cells were divided into five groups: NC group (normal control), Empty vector control group (OE-NC), TMEM71 overexpression group (OE-TMEM71), OE-TMEM71 + si-NC group, and OE-TMEM71 + si-NLRP3 group. Transfection were performed using the Lipo8000™ transfection reagent (Shanghai Beyotime Co.) according to the protocol of manufacturer. All plasmids were synthesized by Sangon Biotech (Shanghai, Co., Ltd.). The sequences of the plasmids used in this study are shown in Table 1.

**Table 1:** Clinical characteristics of 421 patients with nasopharyngeal carcinoma.

Name of plasmid or primer	Sequences (5ʹ–3ʹ)
OE-TMEM71plasmid (F)	TACCGAGCTCGGATCCGCCACCATGTACCGAATATCTCAAC
OE-TMEM71plasmid (R)	GATATCTGCAGAATTCTCAAATTTTGACAAACCGAG
si-NLRP3 (siRNA)	AGAACTAGTTGACTATATA
si-NC plasmid (siRNA)	GCGCGCTTTGTAGGATTCG
TMEM71-F	ACTCATTTATACCAGGAAACAGACA
TMEM71-R	TGTCTGTTTCCTGGTATAAATGAGT
NLRP3-F	CCAGAACCTGCTGTCTTGTG
NLRP3-R	AGAAGGGGTAGCAGTGGTCA
Caspase-1-F	CCTCGCCTTTGCCGATCC
Caspase-1-R	GGATCTTCATGAGGTAGTCAGTC
GSDMD-F	GCCTCCACAACTTCCTGACAGATG
GSDMD-R	GGTCTCCACCTCTGCCCGTAG
β-actin-F	GGAGATTACTGCCCTGGCTCCTA
β-actin-R	GACTCATCGTAC TCCTGCTTGCTG

### qPCR

Total RNA was extracted from cells in their logarithmic growth phase using Trizol reagent (Thermo Fisher Scientific, Shanghai, China). The RNA was then reverse transcribed into cDNA using a reverse transcription kit (Thermo Fisher Scientific). For qPCR, 1 μL of cDNA was used in a 50 μL reaction mix containing SYBR Green dye (Thermo Fisher Scientific) and gene primers. The thermal cycling conditions were initial denaturation at 94 °C for 1 min, followed by 35 cycles of denaturation at 95 °C for 25 seconds, annealing at 62 °C for 30 seconds, and extension at 72 °C for 20 seconds. Gene expression levels were quantified using the 2^−ΔΔCt^ method, with β-actin as the internal control, and primer sequences detailed in Table 1.

### Western blot

Total proteins from HNE-2 cells using RIPA buffer (Thermo Fisher Scientific), and protein concentration was measured by BCA assay (Thermo Fisher Scientific). Equal amounts of protein (30 μg) were separated by SDS-PAGE and transferred onto a PVDF membrane (Beyotime). After blocking with 5% non-fat milk for 1.5 h, the membrane was incubated overnight at 4 °C with primary antibodies: TMEM71 (1:1000, Abcam), NLRP3 (1:1000, Abcam), Caspase-1-p20 (1:1000, Abcam), Pro-Caspase-1 (1:1000, Abcam), GSDMD-N (1:1000, Abcam), and β-actin (1:5000, Abcam). Following washing, the membrane was incubated with HRP-conjugated secondary antibodies (1:5000, Abcam) for 1 h. Protein bands were detected using ECL reagent (Abcam) and visualized with a gel imaging system (Thermo Fisher Scientific). Band intensities were quantified using ImageJ software, and expression levels were normalized to β-actin.

### Co-immunoprecipitation (Co-IP) assay

Total protein from HNE-2 cells was extracted and incubated overnight at 4 °C with IgG and specific antibodies. The following day, protein A/G PLUS-agarose beads were added, and the mixture was incubated for 6 h at 4 °C to capture the immune complexes. The complexes were then dissociated using SDS sample buffer and boiled for 5 min. Western blotting was performed, and the precipitated protein complexes were detected using anti-TMEM71 and anti-NLRP3 antibodies.

### Cell counting kit (CCK)-8 assay

Cells were seeded in a 96-well plates (2 × 10^3^ cells/well) and cultured for 0 h, 24 h, 48 h, and 72 h. Every 24 h, 10 μL of CCK-8 solution (Beyotime) was added and incubated for 2 h at 37 °C in a 5% CO_2_. Absorbance was then measured at 450 nm using a microplate reader (Thermo Fisher Scientific).

### Clonogenic assay

Treated cells were seeded (500 cells/well) in a 6-well plate and incubated for 14 days at 37 °C in 5% CO_2_, with the medium changed every three days. Colonies were then fixed, stained, and counted under a microscope.

### Transwell assay

Cells were suspended in serum-free RPMI 1640 medium and added to the upper chamber of a Transwell insert. The lower chamber was filled with medium containing 10% FBS. After 24 h, non-invading cells were removed, and invading cells in the lower chamber were fixed, stained with crystal violet (0.5%), and counted under a microscope.

### Statistical analysis

Data analysis and graph production were performed using GraphPad Prism (v9.0) and R (v4.3.0) software. Independent t-tests or ANOVA were used for normally distributed data, and the Wilcoxon rank-sum test or Kruskal–Wallis test for non-normal data. Pearson's correlation analysis assessed relationships between variables. Survival curves were generated using the Kaplan–Meier method, with the log-rank test was used for comparisons. Multivariate Cox regression analysis was conducted to identify independent prognostic factors, adjusting for significant variables from univariate analysis. A *p*-value of < 0.05 was considered statistically significant.

## Results

### Acquisition of NRGs in NPC

**Fig. 1:**
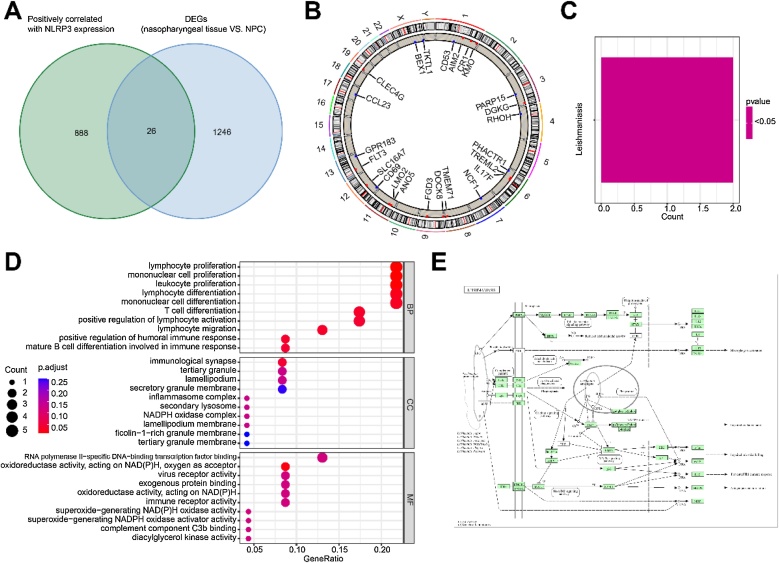
(A) Venn diagram showing the common genes between those positively correlated with NLRP3 expression and differentially expressed genes between normal nasopharyngeal and NPC tumor tissues. (B) Distribution of NLRP3-Related Genes (NRGs) on human chromosomes. (C‒E) KEGG enrichment analysis (C, E) and GO enrichment analysis (D) of the NRGs.

In the GSE53819 dataset, we identified 914 genes positively correlated with NLRP3 expression and 1272 genes downregulated in NPC tumor tissues compared to normal tissues. The intersection of these findings yielded 26 NRGs (Fig. 1A), with their chromosomal locations shown in Fig. 1B. KEGG enrichment analysis linked these NRGs significantly with the leishmaniasis pathway (Fig. 1 C‒E), while GO enrichment analysis highlighted their involvement in immune cell behaviors such as lymphocyte proliferation and differentiation, and notably, in the inflammasome complex (Fig. 1D). Overall, we have successfully identified 26 NRGs.

### Identification of TMEM71 as a hub NRG in NPC

**Fig. 2:**
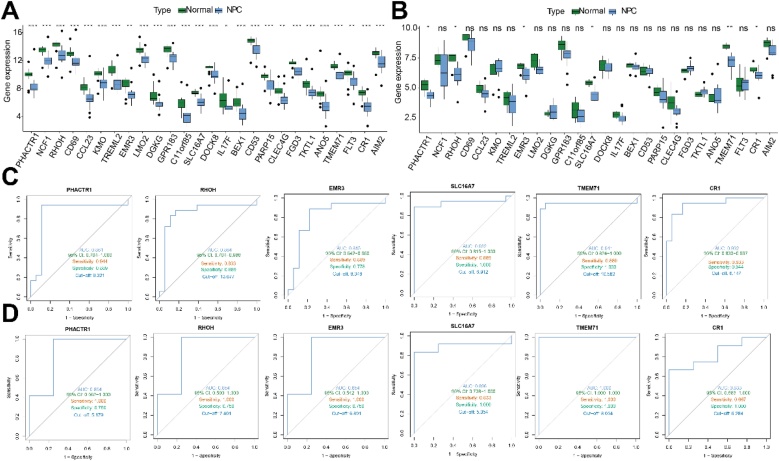
(A‒B) Box plots illustrating the expression differences between tumor and normal tissues for 26 NRGs in the GSE53819 (A) and GSE64634 (B) datasets. (C‒D) ROC curve analyses evaluating the diagnostic value of 6 NRGs for NPC in the GSE53819 (C) and GSE64634 (D) datasets.

**Fig. 3:**
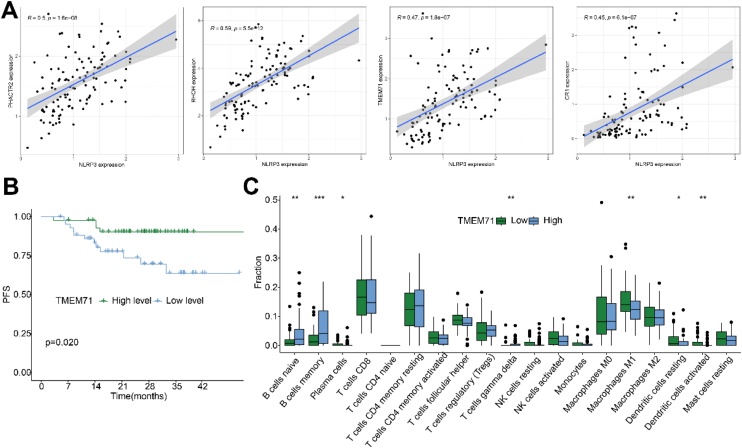
(A) Linear correlation plot illustrating the relationship between NRGs and NLRP3 expression in the GSE102349 dataset. (B) Kaplan–Meier survival curves showing the difference in Progression-Free Survival (PFS) between patients with high and low TMEM71 expression. (C) Box plot showing differences in immune cell infiltration abundance between high and low TMEM71 expression groups in the GSE102349 dataset.

We validated the expression trends of 26 NRGs in the GSE64634 dataset, identifying PHACTR1, RHOH, EMR3, SLC16A7, TMEM71, and CR1 as consistently downregulated in tumor tissues across both GSE64634 and GSE53819 datasets (Fig. 2A‒B). ROC analysis revealed TMEM71 as the most diagnostically promising gene (AUC = 0.941 and 1.000), while EMR3 exhibited the lowest AUC values (0.815 and 0.854) (Fig. 2 C‒D). Six NRGs did not correlate with NLRP3 in the GSE64634 dataset (data not shown). However, further analysis using the GSE102349 revealed that the expression levels of PHACTR1, RHOH, TMEM71, and CR1 were positively correlated with NLRP3 expression (Fig. 3A). TMEM71 was the only gene with significant prognostic value, as higher expression correlated with better PFS (Fig. 3B). Immune cell infiltration analysis revealed significant differences between high and low TMEM71 expression groups, affecting seven immune cell types, such as naïve B-cells and plasma cells (Fig. 3C). In total, TMEM71 is identified as a hub NRG in NPC, with diagnostic, prognostic, and immune modulation potential.

### TMEM71 act as an independent prognostic factor in NPC

**Fig. 4:**
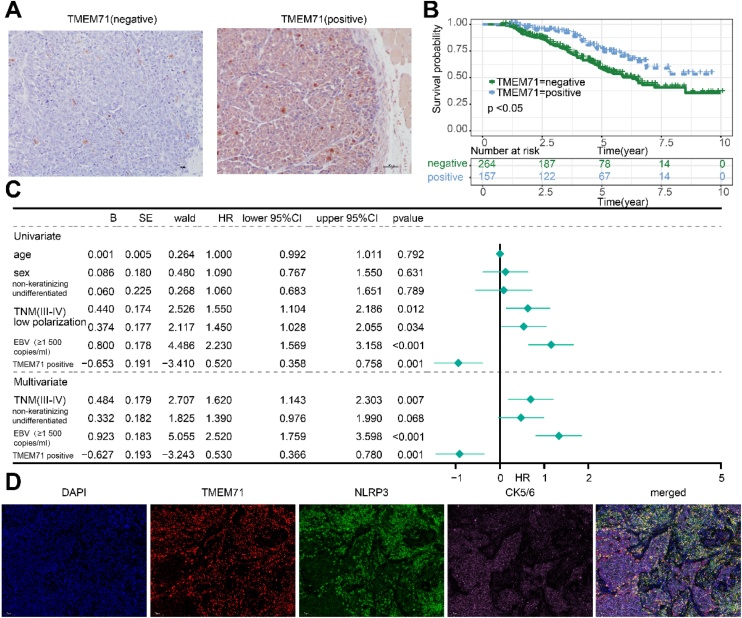
(A) Immunohistochemical staining of TMEM71 in NPC, showing positive and negative expressions (×20 magnification). (B) Kaplan–Meier survival curves depicting the difference in Overall Survival (OS) between patients with high and low TMEM71 expression. (C) Univariate and multivariate Cox regression analyses assessing the prognostic impact of TMEM71 in NPC. (D) Immunofluorescence staining shows the co-localization of TMEM71 and NLRP3 in the cytoplasm of NPC tumor cells.

To investigate the potential role of TMEM71 in NPC, we analyzed local clinical data from 421 NPC patients (Table 2). IHC analysis showed representative images of both positive and negative TMEM71 expression in NPC tumor tissues (Fig. 4A). Kaplan–Meier survival analysis demonstrated that patients with positive TMEM71 expression had significantly better Overall Survival (OS) than those with negative expression (Fig. 4B). Multivariate Cox regression analyses confirmed that positive TMEM71 expression in tumor tissues serves as an independent prognostic factor for NPC (Fig. 4C). Additionally, multiplex immunofluorescence staining revealed co-localization of TMEM71 with NLRP3 in NPC tumor cells (Fig. 4D). These findings highlight the tumor-suppressive potential of TMEM71 and its likely interaction with the NLRP3 in NPC pathogenesis.

**Table 2:** Primer sequences of genes analyzed.

Clinical features	
Age at diagnosis (mean ± SD, years)	51.19 ± 17.01
Gender (n, Male/Female)	262/159
Pathological type (n, non-keratinizing differentiated / non-keratinizing undifferentiated)	71/350
TNM Stage (n, I‒II/III‒IV)	211/210
Tumor cell differentiation (n, low /middle and high polarization)	237/187
EBV load (n, </≥1500 copies/mL)	218/203
TMEM71 (n, negative/positive)	264/157

### TMEM71 may inhibit tumor growth by activating the NLRP3/Caspase-1/GSDMD pathway in NPC

**Fig. 5:**
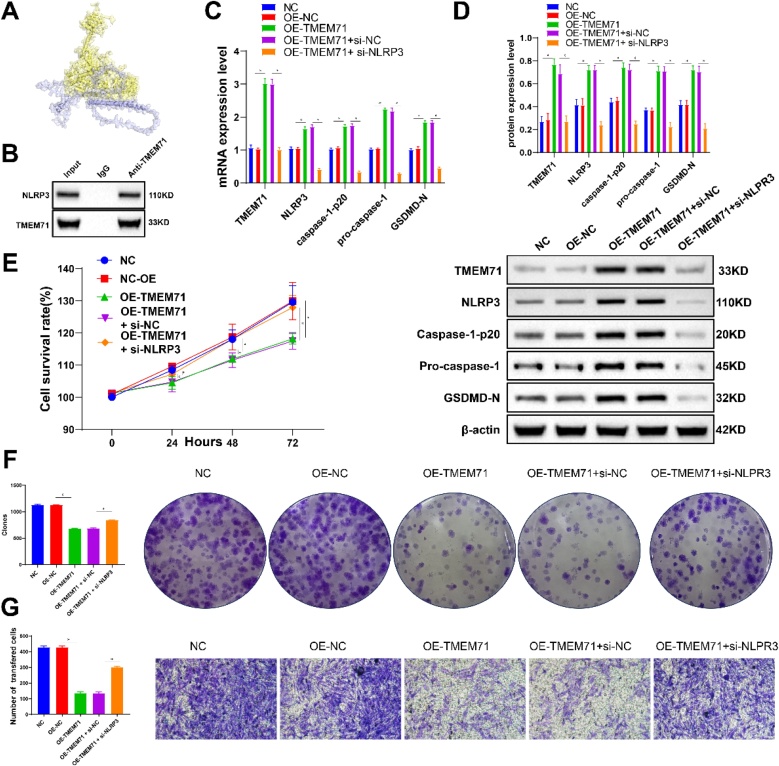
(A) Molecular docking predicts the binding interaction between TMEM71 and NLRP3, with TMEM71 represented in yellow and NLRP3 in blue. (B) Co-immunoprecipitation confirms the interaction between TMEM71 and NLRP3. (C‒D) Expression differences of various genes at the mRNA (C) and protein (D) levels among different groups. (E) CCK-8 assay showing cell viability differences at 0 h, 24 h, 48 h, and 72 h in different cell lines. (F‒G) Clonogenic assay (F) and Transwell assay (G) demonstrating the clonogenic and invasion capabilities of different cell lines.

Molecular docking analysis revealed binding sites between TMEM71 and NLRP3 (Fig. 5A), which was confirmed by co-immunoprecipitation (Fig. 5B). After transfecting with OE-TMEM71 plasmid, activation of the NLRP3/Caspase-1/GSDMD pathway was observed, as confirmed by qPCR and Western blot (Fig. 5C and D). CCK-8 assays showed a significant reduction in NPC cell viability at 24 h, 48 h, and 72 h post-transfection (Fig. 5E), while clonogenic and invasion assays demonstrated reduced cell proliferation and invasiveness following TMEM71 overexpression (Fig. 5F and G). These effects were reversed upon transfection with si-NLRP3 (Fig. 5B‒G). These findings suggest that TMEM71 inhibits NPC tumor growth by interacting with NLRP3 and activating the NLRP3/Caspase-1/GSDMD pathways.

## Discussion

NLRP3, a key regulator of inflammation and pyroptosis, plays a significant role in tumorigenesis.^15^ However, its specific role in NPC remains unclear.^16^, ^17^ Our study identified 26 genes positively correlated with NLRP3 and downregulated in NPC tissues using the GSE53819 dataset, with six genes showing consistent diagnostic value in the GSE64634 dataset. Further analysis using the GSE102349 dataset revealed that TMEM71 is positively correlated with NLRP3 expression and is associated with improved PFS in NPC patients. Data from 421 local NPC patients confirmed TMEM71 as an independent prognostic factor for OS. Molecular docking and co-immunoprecipitation validated the interaction between TMEM71 and NLRP3, while multiplex immunofluorescence showed co-localization of these proteins in tumor cells. In vitro experiments showed that TMEM71 activates the NLRP3/Caspase-1/GSDMD pathway, reducing NPC cell viability, proliferation, and invasiveness. These results indicate the role of TMEM71 in NPC pathogenesis and its relationship with NLRP3.

Recent studies increasingly employ bioinformatics to identify crucial genes in tumor progression. Utilizing TCGA data, previous studies have explored the roles of pyroptosis-related genes in NPC classification and diagnosis, as well as hypoxia-related genes' impact on prognosis, immunotherapy, and cellular communication in head and neck squamous cell carcinoma.^18^, ^19^ Building on this work, our study utilized public transcriptomic databases to focus on NLRP3, a core pyroptosis gene, and its associated genes, providing a detailed representation of pyroptosis-related genes. Ultimately, TMEM71 was identified as the hub NRGs. Recognizing the tumor microenvironment's critical role in cancer progression,^20^ we also explored the relationship between TMEM71 and immune cell infiltration using the ssGSEA algorithm. Results indicated a positive correlation between TMEM71 expression and B cell infiltration, consistent with prior findings that higher B-cell density correlates with better NPC prognosis.^21^ Although previous studies suggest a tumor-suppressive role for M1 macrophages^22^, ^23^ and dendritic cells^24^, ^25^ in NPC, our analysis revealed a negative correlation between TMEM71 and these immune cells, highlighting disease heterogeneity and the immune complexity. This suggests that TMEM71 may regulate immune cell infiltration and cell-cell communication, warranting further exploration. Additionally, our findings on the negative correlation between TMEM71 and plasma cells could provide new research directions, as the role of plasma cells in NPC remains unclear.

The role of TMEM71 in various cancers is garnering attention. In gliomas, elevated TMEM71 expression correlates with enhanced immune responses, while in breast cancer, reduced TMEM71 levels have been noted, with studies showing its overexpression can curb cell proliferation and migration.^26^, ^27^ TMEM71 is also a component of a prognostic model for papillary renal cell carcinoma.^28^ However, its role in NPC remains underexplored. Our study marks the first to outline TMEM71's tumor-suppressive impact in NPC, establishing its positive expression as a standalone protective factor for overall survival. We found that TMEM71 curtails malignant behaviors in NPC cell lines and uniquely activates the NLRP3/Caspase-1/GSDMD pathway. Other TMEM family highlight their broad implications in cancer dynamics. For instance, TMEM25 inhibits EGFR-driven STAT3 activation in triple-negative breast cancer, and TMEM116 is crucial for lung cancer cell mobility and metastasis via the PDK1 pathway.^29^, ^30^ TMEM9A promotes breast cancer progression by activating the Wnt/β-Catenin pathway.^31^ Overall, TMEM proteins influence cell proliferation, epithelial-mesenchymal transition, invasion, migration, and immune modulation, highlighting their potential as therapeutic targets in cancer.^32^

Although our study is the first to reveal the anti-tumor role of TMEM71 in NPC, several limitations exist. We did not conduct animal experiments, which would clarify mechanisms, particularly in the immune microenvironment. The use of bulk transcriptome data limits cells specific insights, potentially affecting the reliability of observed expression trends. Additionally, retrospective analysis of clinical data may introduce selection bias, and we did not compare TMEM71 with other established NPC diagnostic markers like EBV DNA. Despite these limitations, this study fills the knowledge gap regarding the role of NLRP3 in NPC progression, suggesting that TMEM71, possibly through the NLRP3 pathway, could become a novel therapeutic target. Future animal models, single-cell analyses, prospective studies, and diagnostic comparisons will be essential to validate our findings and explore therapeutic potential.

## Conclusions

TMEM71 is downregulated in NPC tissues and significantly associated with PFS, OS, and immune cell infiltration. Overexpression of TMEM71 activates the NLRP3/Caspase-1/GSDMD pathway, inhibiting NPC cell malignancy. These findings indicate TMEM71's potential as a prognostic marker, as well as a therapeutic target in NPC.

## Funding

This work was supported by the Hubei Provincial Natural Science Foundation of China (grant nº 2022CFB498).

## Declaration of competing interest

The authors declare no conflicts of interest.

## References

[1] Chen Y.P., Chan A.T.C., Le Q.T. (2019). Nasopharyngeal carcinoma. Lancet (London, England).

[2] Almobarak A.A., Jebreel A.B., Abu-Zaid A. (2019). Molecular targeted therapy in the management of recurrent and metastatic nasopharyngeal carcinoma: a comprehensive literature review. Cureus.

[3] Qu W.L., Li S.H., Zhang M. (2020). Pattern and prognosis of distant metastases in nasopharyngeal carcinoma: a large-population retrospective analysis. Cancer Med..

[4] Matt L., Volker H.S., Christopher D.S. (2021). Somatostatin receptor 2 expression in nasopharyngeal cancer is induced by Epstein Barr virus infection: impact on prognosis, imaging and therapy. Nat Commun..

[5] Wei X., Yu S., Wang J. (2024). Association between time from diagnosis to treatment and survival of patients with nasopharyngeal carcinoma: a population-based cohort study. Curr Probl Cancer.

[6] Li M.Y., Jiang P., Yang Y. (2023). The role of pyroptosis and gasdermin family in tumor progression and immune microenvironment. Exp Hematol Oncol..

[7] He Y.M., Jiang S.Y., Cui Y.L. (2024). Induction of IFIT1/IFIT3 and inhibition of Bcl-2 orchestrate the treatment of myeloma and leukemia via pyroptosis. Cancer Lett..

[8] Zhu H.Y., Guan Y.F., Wang W. (2024). Reniformin A suppresses non-small cell lung cancer progression by inducing TLR4/NLRP3/caspase-1/GSDMD-dependent pyroptosis. Int Immunopharmacol..

[9] Liu D., Wan L., Peng C. (2023). [Expressions of NLRP3, Caspase-1, and GSDMD in nasopharyngeal carcinoma tissue and association with recurrence and metastasis]. Zhonghua Er Bi Yan Hou Tou Jing Wai Ke Za Zhi..

[10] Li Z., Guo Z., Xiao H.T. (2023). Simulating neuronal development: exploring potential mechanisms for central nervous system metastasis in acute lymphoblastic leukemia. Front. Oncol..

[11] Wu Q.R., Yang H., Zhang H.D. (2024). IP3R2-mediated Ca(2+) release promotes LPS-induced cardiomyocyte pyroptosis via the activation of NLRP3/Caspase-1/GSDMD pathway. Cell death Discov..

[12] Kang H., Lee C.J. (2024). Transmembrane proteins with unknown function (TMEMs) as ion channels: electrophysiological properties, structure, and pathophysiological roles. Exp Mol Med..

[13] Zhang H., Zeng L., Xie M. (2020). TMEM173 drives lethal coagulation in sepsis. Cell Host Microbe.

[14] Wu R., Wang N., Comish P.B. (2021). Inflammasome-dependent coagulation activation in sepsis. Front Immunol..

[15] Chao L., Zhang W., Feng Y. (2024). Pyroptosis: a new insight into intestinal inflammation and cancer. Front Immunol..

[16] Li Q., Wang M., Zhang Y. (2020). BIX-01294-enhanced chemosensitivity in nasopharyngeal carcinoma depends on autophagy-induced pyroptosis. Acta Biochim Biophys Sin (Shanghai)..

[17] Wang X., Li H.Q., Li W. (2020). The role of Caspase-1/GSDMD-mediated pyroptosis in Taxol-induced cell death and a Taxol-resistant phenotype in nasopharyngeal carcinoma regulated by autophagy. Cell Biol Toxicol..

[18] Wang Y., Zou Y.X., Chen X.H. (2024). Relevance of pyroptosis-associated genes in nasopharyngeal carcinoma diagnosis and subtype classification. J Gene Med..

[19] Peng C., Ye H.P., Li Z.Y. (2023). Multi-omics characterization of a scoring system to quantify hypoxia patterns in patients with head and neck squamous cell carcinoma. J Transl Med..

[20] Benmelech S., Le T., McKay M. (2024). Biophysical and biochemical aspects of immune cell-tumor microenvironment interactions. APL Bioeng..

[21] Chen C., Zhang Y., Wu X. (2024). The role of tertiary lymphoid structure and B cells in nasopharyngeal carcinoma: Based on bioinformatics and experimental verification. Transl Oncol..

[22] Chen W., Bao L.L., Ren Q.Q. (2023). SCARB1 in extracellular vesicles promotes NPC metastasis by co-regulating M1 and M2 macrophage function. Cell Death Discov..

[23] Chen P., Wang D., Xiao F.T. (2023). ACSL4 promotes ferroptosis and M1 macrophage polarization to regulate the tumorigenesis of nasopharyngeal carcinoma. Int Immunopharmacol..

[24] Wang H., Huang S.P., Wu S.Y. (2017). Follistatin-like protein-1 upregulates dendritic cell-based immunity in patients with nasopharyngeal carcinoma. J Interf Cytokine Res Off J Int Soc Interf Cytokine Res..

[25] Nickles E., Dharmadhikari B., Yating L. (2022). Dendritic cell therapy with CD137L-DC-EBV-VAX in locally recurrent or metastatic nasopharyngeal carcinoma is safe and confers clinical benefit. Cancer Immunol Immunother..

[26] Wang K.Y., Huang R.Y., Tong X.Z. (2019). Molecular and clinical characterization of TMEM71 expression at the transcriptional level in glioma. CNS Neurosci Ther..

[27] Li W., Wang X., Li C. (2023). Identification and validation of an m6A-related gene signature to predict prognosis and evaluate immune features of breast cancer. Hum Cell.

[28] Liu Z., Wan Y., Yang M. (2020). Identification of methylation-driven genes related to the prognosis of papillary renal cell carcinoma: a study based on The Cancer Genome Atlas. Cancer Cell Int..

[29] Mohan C.D., Rangappa K.S., Sethi G. (2024). Transmembrane protein 25 abrogates monomeric EGFR-driven STAT3 activation in triple-negative breast cancer. MedComm.

[30] Zhang S., Dai H., Li W. (2021). TMEM116 is required for lung cancer cell motility and metastasis through PDK1 signaling pathway. Cell Death Dis..

[31] He J., Bu Y., Li X. (2023). Tumor-promoting properties of TMEM9A in breast cancer progression via activating the Wnt/β-Catenin signaling pathway. Biol Pharm Bull..

[32] Herrera-Quiterio G.A., Encarnación-Guevara S. (2023). The transmembrane proteins (TMEM) and their role in cell proliferation, migration, invasion, and epithelial-mesenchymal transition in cancer. Front Oncol..

